# PISCOM: a new procedure for epilepsy combining ictal SPECT and interictal PET

**DOI:** 10.1007/s00259-018-4080-6

**Published:** 2018-08-01

**Authors:** Andrés Perissinotti, Aida Niñerola-Baizán, Sebastià Rubí, Mar Carreño, Berta Marti-Fuster, Javier Aparicio, Maria Mayoral, Antonio Donaire, Nuria Sanchez-Izquierdo, Nuria Bargalló, Jordi Rumiá, Teresa Boget, Francesca Pons, Francisco Lomeña, Domènec Ros, Javier Pavía, Xavier Setoain

**Affiliations:** 1 Department of Nuclear Medicine, Hospital Clínic, C/Villarroel 170, 08036 Barcelona, Spain; 2 Biomedical Research Networking Center in Bioengineering, Biomaterials and Nanomedicine (CIBER-BBN), Barcelona, Spain; 3 University of Barcelona, Barcelona, Spain; 4 Nuclear Medicine Department, Hospital Universitari Son Espases, Palma, Spain; 5 Institut d’Investigació Sanitària Illes Balears (IdISBa), Palma, Spain; 6 Department of Neurology, Hospital Clínic, Barcelona, Spain; 7 Department of Neurology, Hospital Sant Joan de Déu, Barcelona, Spain; 8 Department of Radiology, Hospital Clínic, Barcelona, Spain; 9 Department of Neurosurgery, Hospital Clínic, Barcelona, Spain; 10 Department of Psychiatry and Psychology, Hospital Clínic, Barcelona, Spain

**Keywords:** PISCOM, SISCOM, Functional neuroimaging, PET in epilepsy, SPECT in epilepsy

## Abstract

**Purpose:**

We present a modified version of the SISCOM procedure that uses interictal PET instead of interictal SPECT for seizure onset zone localization. We called this new nuclear imaging processing technique PISCOM (PET interictal subtracted ictal SPECT coregistered with MRI).

**Methods:**

We retrospectively studied 23 patients (age range 4–61 years) with medically refractory epilepsy who had undergone MRI, ictal SPECT, interictal SPECT and interictal FDG PET and who had been seizure-free for at least 2 years after surgical treatment. FDG PET images were reprocessed (rFDG PET) to assimilate SPECT features for image subtraction. Interictal SPECT and rFDG PET were compared using statistical parametric mapping (SPM). PISCOM and SISCOM images were evaluated visually and using an automated volume of interest-based analysis. The results of the two studies were compared with each other and with the known surgical resection site.

**Results:**

SPM showed no significant differences in cortical activity between SPECT and rFDG PET images. PISCOM and SISCOM showed equivalent results in 17 of 23 patients (74%). The seizure onset zone was successfully identified in 19 patients (83%) by PISCOM and in 17 (74%) by SISCOM: in 15 patients (65%) the two techniques showed concordant successful results. The volume of interest-based analysis showed no significant differences between PISCOM and SISCOM in identifying the extension of the seizure onset zone. However, PISCOM showed a lower amount of indeterminate activity due to propagation, background or artefacts.

**Conclusion:**

Preliminary findings of this initial proof-of-concept study suggest that perfusion and glucose metabolism in the cerebral cortex can be correlated and that PISCOM may be a valid technique for identification of the seizure onset zone. However, further studies are needed to validate these results.

**Electronic supplementary material:**

The online version of this article (10.1007/s00259-018-4080-6) contains supplementary material, which is available to authorized users.

## Introduction

The success of surgical treatment of drug-resistant epilepsy is determined by the accuracy of presurgical identification of the epileptogenic zone (EZ) defined as “the minimum amount of cortex that must be resected to produce seizure freedom” [1]. The subtraction of interictal SPECT from the ictal SPECT coregistered with MRI (SISCOM) technique for evaluation of the seizure onset zone (SOZ) was first described by O’Brien et al. [2]. SISCOM improves SPECT sensitivity and specificity in the presurgical identification of the SOZ with higher rates of favourable postoperative outcomes [3–11]. SISCOM requires the acquisition of both ictal and interictal perfusion SPECT studies. Ictal SPECT is able to detect the increase in regional cerebral blood flow related to the SOZ while interictal SPECT shows hypoperfusion or normal perfusion in the dysfunctional brain areas related to the EZ. However, the role of interictal SPECT has been relegated as a baseline study for ictal SPECT subtraction due to its lower sensitivity [9, 12–15].

Interictal dysfunctional brain areas related to the EZ are also represented by ^18^F-fluorodeoxyglucose (FDG) PET as hypometabolic glucose-consuming regions that do not necessarily match the SOZ. Nonetheless, FDG PET offers higher resolution and a better signal-to-noise ratio than SPECT, providing valuable information that can modify surgical management and improve intracranial electrode placement [6, 8, 9, 16, 17]. FDG PET has been demonstrated to be more sensitive and accurate than interictal SPECT, usually depicting a larger dysfunctional hypometabolic brain area than perfusion SPECT [9, 13, 15, 18–25].

By subtracting ictal from interictal perfusion studies, SISCOM has demonstrated that variations in ictal/interictal perfusion can lead to successful SOZ identification. On the basis of the idea that ictal hyperperfusion originates somewhere within the broader dysfunctional hypometabolic areas [10], the purpose of this work was to determine if variations between ictal perfusion and interictal glucose metabolism could also lead to successful identification of the SOZ. We hypothesized that interictal FDG PET could be used as a valid baseline study for subtraction from ictal SPECT replacing interictal SPECT in the SISCOM procedure.

This study was an initial proof-of-concept study in the development of a new image processing technique consisting of interictal FDG PET images subtracted from ictal perfusion SPECT studies coregistered with MRI (PET interictal subtracted ictal SPECT coregistered with MRI; PISCOM). We compared the results with those of the conventional SISCOM technique for the presurgical localization of the SOZ.

## Materials and methods

A more detailed version of methodological aspects, especially regarding SPECT and FDG PET acquisition, reconstruction, processing and image subtraction procedures, are set out in the Supplementary material.

### Patients

We performed a retrospective review of all patients with medically refractory epilepsy who had undergone presurgical evaluation and subsequent surgical resection at our centre from 2008 to 2013. The inclusion criteria were: (a) diagnosis of medically refractory epilepsy, (b) patient had undergone brain MRI, ictal SPECT, interictal SPECT and FDG PET studies, (c) surgical resection of the suspected EZ, and (d) favourable outcome (Engel class I or II) for at least 2 years after surgery. The patients had been previously evaluated in the clinical work-up to optimally localize the EZ using clinical and neuropsychological examinations, EEG monitoring, and anatomical and functional neuroimaging studies, as described in a previously [8]. On the basis of the inclusion criteria, 23 patients with a mean age of 31 years (range 4–61 years) were retrospectively identified (10 male and 13 female, 18 adults and 5 children). Informed written consent was obtained from all patients or their parents or legal guardians, and all procedures were approved by the hospital Ethics Committee.

### Surgical treatment and histological analysis

Patient candidacy for surgery was established based on the standard evaluation protocol of the epilepsy unit according to the clinical, electrophysiological, and neuroimaging data obtained. Lobar or selective cortical resection was performed by neurosurgeons from the epilepsy unit. Histological analysis of all the brain tissue resected showed that 9 of the 23 patients (39%) had mesial temporal sclerosis, 9 (39%) had focal cortical dysplasia, two had mesial temporal sclerosis associated with focal cortical dysplasia, one had gliosis, one had low-grade glioma and one had grade I ganglioglioma (Table 1).

**Table 1:** Epileptogenic zone location, SISCOM, PISCOM and MRI findings, surgical resection, histology results, ictal SPECT injection time to seizure, follow-up time and surgical outcome in all the patients studied

Patient no.	Age (years)	Sex	Epileptogenic zone location^a^	SISCOM		PISCOM		MRI^c^	Surgical resection	Histology	Injection time (s)	Follow-up (years)	Engel class
				Result	Identification success^b^	Result	Identification success^b^						
P01	32	M	Right MT	Right MT	Successful	Right MT and pole	Successful	Right MTS with pole compromise	Right ATL	MTS	36	2	I
P02	61	F	Left MT and LT	Left LT	Successful	Left T pole	Successful	Left MTS	Left ATL	MTS + FCD IIIa	15	4	I
P03	33	M	Left MT	Left T pole	Successful	Left T pole	Successful	Left MTS with pole compromise	Left ATL	MTS	23	5	I
P04	46	F	Right MT and LT posterior	Right T pole	Successful	Right T pole	Successful	Right MTS with pole compromise and posterior dysplasia	Right ATL and LT posterior	MTS + FCD IIIa	25	2	I
P05	35	M	Right LT	Right T pole	Successful	Right T pole	Successful	Right P cavernoma	Right T lateral	Gliosis	19	2	I
P06	30	M	Right MT	Right T pole	Successful	Right T pole	Successful	Right MTS	Right ATL	MTS	34	4	I
P07	56	M	Right MT	Right MT	Successful	Right T pole	Successful	Right MTS	Right ATL	MTS	14	2.5	I
P08	41	F	Right MT	Negative		Right T pole	Successful	Right MTS	Right ATL	MTS	17	3	I
P09	50	F	Left MT	Left T pole	Successful	Left T pole	Successful	Left MTS	Left ATL	MTS	18	5	I
P10	25	F	Right F posterior	Right F posterior	Successful	Right F posterior	Successful	Negative	Right F partial lobectomy	FCD II	8	2	I
P11	23	F	Right MT	Right T pole	Successful	Right T pole	Successful	Right MTS	Right ATL	MTS	46	5	I
P12	40	F	Right MT	Right MT	Successful	Right MT	Successful	Negative	Right ATL	MTS	26	3	I
P13	31	F	Left MT	Left MT	Successful	Right MT	Unsuccessful	Left T fusiform gyrus dysplasia	Left T fusiform gyrus	FCD Ia	32	3.5	I
P14	34	F	Left LT posterior	Left MT	Unsuccessful	Left T pole	Unsuccessful	Left T posterior dysplasia or ganglioglioma	Left T posterior	Low-grade glioma	22	4	I
P15	52	F	Right LT	Right T pole	Successful	Right T pole	Successful	Negative	Right ATL	FCD I	23	2.5	I
P16	25	M	Right F superior	Right F superior	Successful	Right MT	Unsuccessful	Right F superior dysplasia	Right F partial lobectomy	FCD	10	2	I
P17	18	M	Right Insula	Left P	Unsuccessful	Right Insula	Successful	Right Insula dysplasia	Right insula	Grade I ganglioglioma	8	2	I
P18	44	F	Right MT	Right T pole	Successful	Right T pole	Successful	Right MTS with pole compromise	Right ATL	MTS	30	3.5	I
P19	11	F	Right F middle	Right F middle	Successful	Right F middle	Successful	Right F middle dysplasia	Right F partial lobectomy	FCD IIa	37	5	I
P20	9	F	Right F inferior	Negative		Right F inferior	Successful	Right F middle dysplasia	Right F partial lobectomy	FCD IIb	18	5	I
P21	4	M	Left F superior	Negative		Negative		Left F superior dysplasia	Left F partial lobectomy	FCD IIa	4	4	I
P22	4	M	Right F	Left MT	Unsuccessful	Right F middle and superior	Successful	Right F superior, orbital and cingulate dysplasia	Right F partial lobectomy	FCD IIa	8	4	I
P23	9	M	Left P inferior	Left P inferior	Successful	Left P inferior	Successful	Left P inferior dysplasia	Left P partial lobectomy	FCD IIb	22	3.5	I

*T* temporal, *F* frontal, *P* parietal, *MT* medial temporal lobe, *LT* lateral neocortical temporal lobe, *MTS* mesial temporal sclerosis, *FCD* focal cortical dysplasia, *ATL* anterior temporal lobectomy

^a^Based on surgical resection, histology and follow-up results

^b^Success in identifying the seizure onset zone

^c^MRI results not blinded to the clinical data and results of other tests

### Follow-up

Postoperative seizure outcome was classified according to the Engel classification. Only patients with a favourable outcome (Engel class I or II) for a period of at least 2 years after surgery were included in the study. All 23 patients were classified as Engel class I with a median follow-up time of 3.41 years (range 2–5 years; Table 1).

### Epileptogenic zone

The EZ was considered the gold standard based on surgical resection, histology results and favourable surgical outcome. The EZ was located in the temporal lobe in 15 of the 23 patients (65%), with 10 patients (43%) having lesions in the medial temporal lobe, 3 (13%) in the lateral neocortical temporal lobe and 2 (9%) in both the medial temporal and the lateral neocortical temporal lobe. Of the remaining 8 patients, the EZ was located in the frontal lobe in 6 (26%), the insula in 1 (4%) and the parietal lobe in 1 (4%; Table 1).

### MRI

In all of 18 adult patients, MRI was performed with a 3-T unit (Tim Trio; Siemens, Erlangen, Germany) and in the 5 paediatric patients with a 1.5-T unit (Signa Exite; GE Healthcare, Milwaukee, WI) with a specific epilepsy protocol. All the MRI studies were interpreted visually by an expert neuroradiologist not blinded to the clinical data or the results of other tests.

### SPECT

Ictal SPECT was performed as part of patient admission for video-EEG monitoring in the epilepsy unit of our centre. Seizure onset was defined as the time of earliest indication of auras or the beginning of rhythmic ictal discharges detected by continuous video-EEG monitoring. The radiotracer was administered intravenously at seizure onset. Interictal SPECT was performed during the week after the patient had been free of seizures for more than 24 h. Ictal and interictal SPECT images were acquired within 2 h of injection of approximately 925 MBq of ^99m^Tc-hexamethylpropyleneamine oxime (^99m^Tc-HMPAO) radiotracer following the same protocol using a dual-head SPECT imaging system (Infinia™ Hawkeye™ 4; GE Healthcare Milwaukee, WI).

### PET

Interictal FDG PET images were acquired in 3D mode using a PET/CT system (Biograph; Siemens, Erlangen, Germany) after intravenous injection of approximately 5 MBq/kg of ^18^F-FDG. In selected cases, EEG monitoring was performed during FDG PET image acquisition to confirm interictal state. SPECT and PET studies were performed within an overall mean period of 86 days (range 7–201 days). No changes in medication or clinical epilepsy features were documented during this period of time.

### SISCOM

SPECT images were reconstructed using iterative algorithms including scatter, attenuation and point spread function (PSF) corrections with the aim of obtaining SPECT images with resolution and degradation corrections more similar to those of FDG PET. Perfusion SPECT subtraction according to SISCOM methodology was performed using FocusDET, a previously developed software toolbox for SISCOM analysis [26].

### PISCOM

**Fig. 1:**
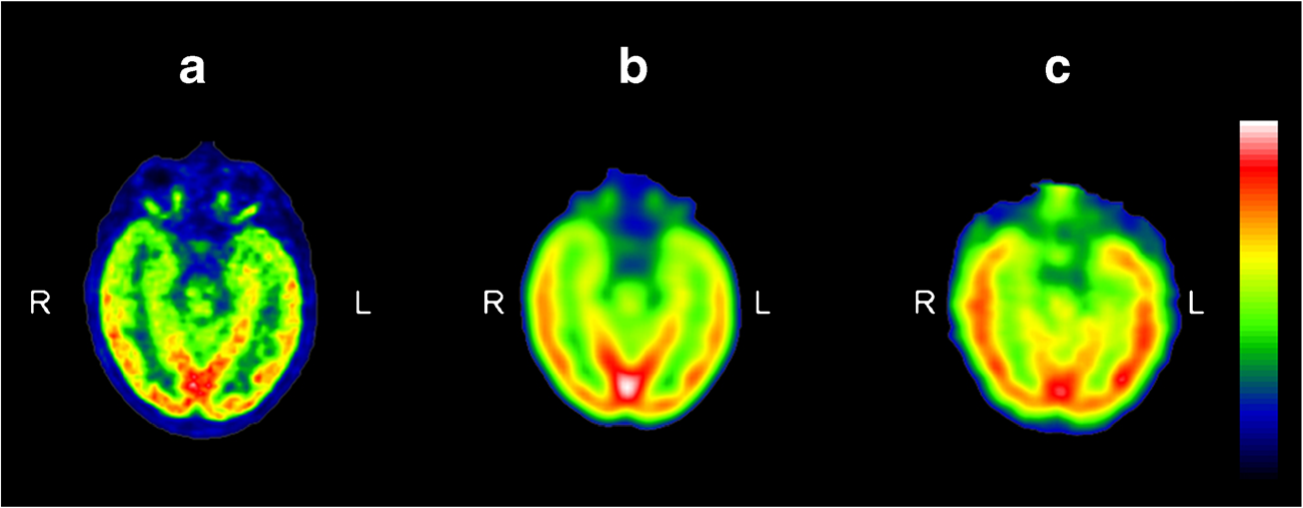
Reprocessed FDG PET images. Axial images: **a** original FDG PET, **b** reprocessed FDG PET (rFDG PET), **c** interictal SPECT. All images are coregistered with MRI images. The *colour scale* indicates intensity values. *L* left, *R* right

With the aim of subtracting interictal PET images from ictal SPECT images, FDG PET images were reprocessed (rFDG PET) to assimilate features of the PET and SPECT images as follows: (1) FDG PET images were resampled to obtain the same matrix and voxel size as those of interictal perfusion SPECT, (2) FDG PET images were filtered to achieve similar smoothing between the SPECT and PET images (the FDG PET filter was derived from experimental PSF images of the SPECT and the PET acquisition systems), and (3) intensity normalization was applied to FDG PET images to correct for differences in the total number of photons detected (Fig. 1). As the loss of resolution that the real image suffered in the acquisition was due to the PSF, the FDG PET filter in step 2 was derived from experimental PSFs obtained from the SPECT and PET systems by using a radioactive point source. Fourier transforms of SPECT and PET PSFs were then calculated and both distributions were fitted to a gaussian distribution to avoid problems derived from high-frequency noise. Finally, the filter was obtained as the inverse Fourier transform of the quotient of the gaussian associated with SPECT and that associated with PET.

The PISCOM method was performed using FocusDET, following the same steps as the SISCOM analysis but replacing the interictal SPECT image with the rFDG PET image.

### SPM analysis (rFDG PET vs. interictal SPECT)

Systematic differences in the inherent biodistributions of ^99m^Tc-HMPAO and ^18^F-FDG were compared between the interictal SPECT and rFDG PET studies using the statistical parametric mapping (SPM8) software (Wellcome Department of Imaging Neuroscience, Institute of Neurology, London) [27]. A paired *t* test was performed (two sample *t* test: group 1, 23 interictal SPECT images; group −1, 23 rFDG PET images) applying two contrast levels [1 –1] and [−1 1]. If any significant differences related to the biodistribution of the radiotracer were detected at a subcortical or cerebellar level, a mask based on these findings was created to exclude these areas from the visual and volume of interest (VOI)-based analysis of the PISCOM images.

### Visual analysis

The PISCOM and SISCOM images were interpreted separately by two experts in nuclear medicine blinded to the clinical data and the results of other tests. PISCOM and SISCOM studies were superimposed on a basic T1 MRI sequence only to provide anatomical reference. In addition, the automated anatomical labelling (AAL) template was used for better anatomical localization of the findings. Abnormal findings were defined by visual evaluation of brain areas with activity greater than two standard deviations above the mean activity. The SOZ was defined as the cortical area with the greatest subtraction values that could not be explained by propagation, residual count data or artefacts. The presence and location of the SOZ and propagation activity with both modalities was reported. Both imaging modalities in each patient were presented randomly to ensure that the review of one study would not influence that of the other. In cases of discrepancy between reviewers, a third nuclear medicine specialist was consulted to achieve consensus.

The PISCOM and SISCOM results were compared with each other and with the EZ location. Successful identification of the SOZ had to match the PISCOM or SISCOM findings with the known EZ location at a sublobar level of accuracy; hemisphere or simply lobar concordance was considered as unsuccessful localization. Nevertheless, a focus located in the temporal pole was considered successful identification of the SOZ regardless of whether the EZ was located in the medial temporal lobe or the lateral neocortical temporal lobe. When PISCOM or SISCOM failed to identify any focus, the study were classified as “negative”. In summary, each PISCOM or SISCOM study was classified as “successful”, “unsuccessful” or “negative”.

### VOI-based analysis

To perform a more objective comparison between the PISCOM and SISCOM results, a supplementary analysis of all 15 concordant successful studies was carried out. The extension of each SOZ was quantified, as well as the amount of indeterminate activity that could hamper the identification of the SOZ with both modalities. First, the maximum value of the subtraction image in each SOZ identified by PISCOM and SISCOM was determined. VOIs were then obtained in each PISCOM and SISCOM study by intensity thresholding (60% of the maximum SOZ value) and clustering (minimum 100 voxels). Thus, each PISCOM and SISCOM study was segmented into several VOIs, one corresponding to the SOZ and the remainder corresponding to VOIs that matched the threshold criteria which were considered as indeterminate activity (i.e. propagation, background or artefact). Specific algorithms were developed for this task. The number of VOIs in each PISCOM and SISCOM study was determined to evaluate the amount of indeterminate activity revealed by the two modalities. In addition, the number of voxels of each VOI corresponding to the SOZ delimited by PISCOM and SISCOM was quantified to compare the SOZ voxel extension in each patient. The percentages of voxels shared by the SOZ from the two modalities were calculated.

### Statistical analysis

The proportions of the PISCOM and SISCOM results that were “successful”, “unsuccessful” and “negative” were calculated and compared (McNemar test of symmetry). The global agreement between the results of the two techniques was calculated. The proportions of successful identification of the SOZ by PISCOM and SISCOM are reported with their 95% confidence intervals (CI). Variables derived from the VOI-based analysis (number of VOIs and SOZ voxel extension) were compared using a paired *t* test (*p* < 0.05 was considered significant). Statistical analyses were performed using SPSS Statistics, version 17.0 (SPSS Inc., Chicago).

## Results

### SPM analysis

**Fig. 2:**
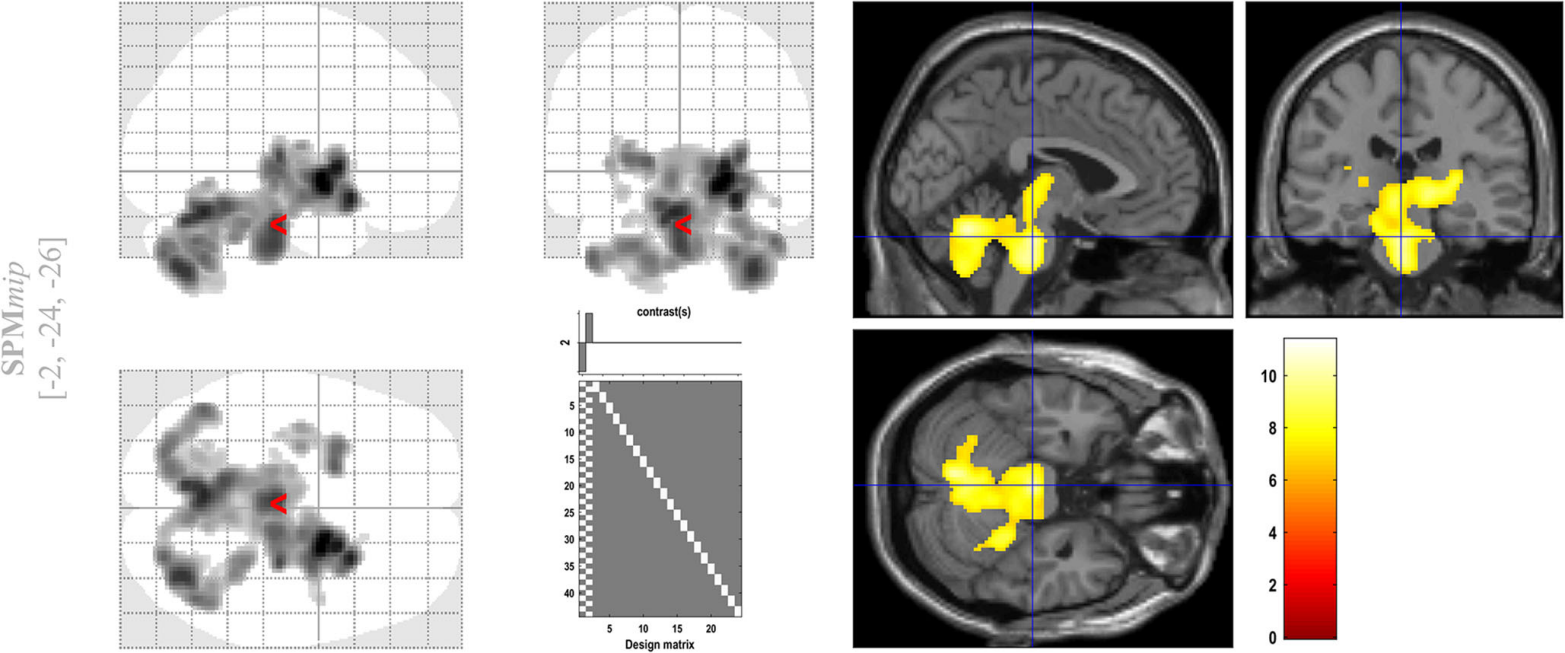
SPM results. SPM paired *t* test comparing interictal reprocessed FDG PET (rFDG PET) and interictal SPECT images showed higher activity on SPECT images with significant differences exclusively in the cerebellum, midbrain and basal ganglia. On the left, significant SPM results are shown in the form of maximum intensity projections (SPMmip) in the sagittal, coronal and axial views. On the right, *t* values obtained from the paired *t* test are superimposed on the MRI image available in SPM. Only significant *t* values are shown (colour scale from red to white)

The paired *t* test comparing interictal SPECT and rFDG PET (contrast [1 –1]) showed no significant differences in cortical activity. However, the SPECT group showed higher activity than the rFDG PET group in the cerebellum, midbrain and basal ganglia (*p* < 0.05, corrected for multiple comparisons using the family-wise error rate; Fig. 2). The paired *t* test comparing rFDG PET and interictal SPECT (contrast [−1 1]) showed no significant cortical or subcortical differences.

### Visual analysis

**Fig. 3:**
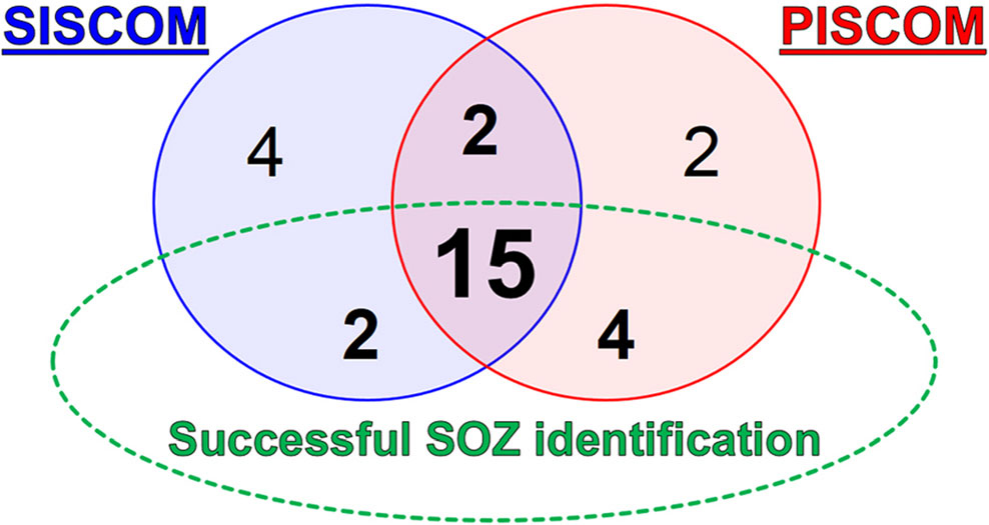
Visual analysis results. Venn diagram showing total number of patients studied by SISCOM and PISCOM, successful identifications of the SOZ and their concordance

PISCOM and SISCOM showed equivalent results in 17 of 23 patients with good concordance (global agreement 74%, 95% CI 56–87%; Fig. 3). PISCOM successfully identified the SOZ in 19 patients (83%) and SISCOM in 17 patients (74%). However, there were no significant differences in the proportions of the PISCOM and SISCOM results that were “successful”, “unsuccessful” and “negative” (*p* = 0.368).

The two techniques showed concordant successful SOZ localization in 15 of the 23 patients (65%). Of the two remaining patients with concordant but unsuccessful localization, the SOZ was located at a lobar level in one (P14), and in the other (P21) PISCOM and SISCOM failed to identify any focus. Temporal lobe epilepsy was successfully identified in 13 of 15 patients (87%) by both PISCOM and SISCOM. Of the eight patients with extratemporal epilepsies, the SOZ was successfully identified by PISCOM in six (75%) and by SISCOM in four (50%). The PISCOM and SISCOM results were discordant in 6 patients (26%). Together, PISCOM or SISCOM successfully identified the SOZ in 21 patients (91%; Table 1 and Fig. 3). Propagation activity was visually observed by PISCOM in 14 patients (61%) and by SISCOM in 15 patients (65%), and in nine of these patients coinciding propagation was observed at a lobar level of concordance. In 19 patients (83%) MRI successfully identified a lesion consistent with the EZ location. In three of the remaining patients (P10, P12 and P15), studies were reported as nonlesional or negative, and in one patient (P05) MRI detected a right parietal cavernoma nonconcordant with the SOZ localization; PISCOM and SISCOM successfully identified the SOZ in all four of these patients (Table 1).

### VOI-based analysis

**Fig. 4:**
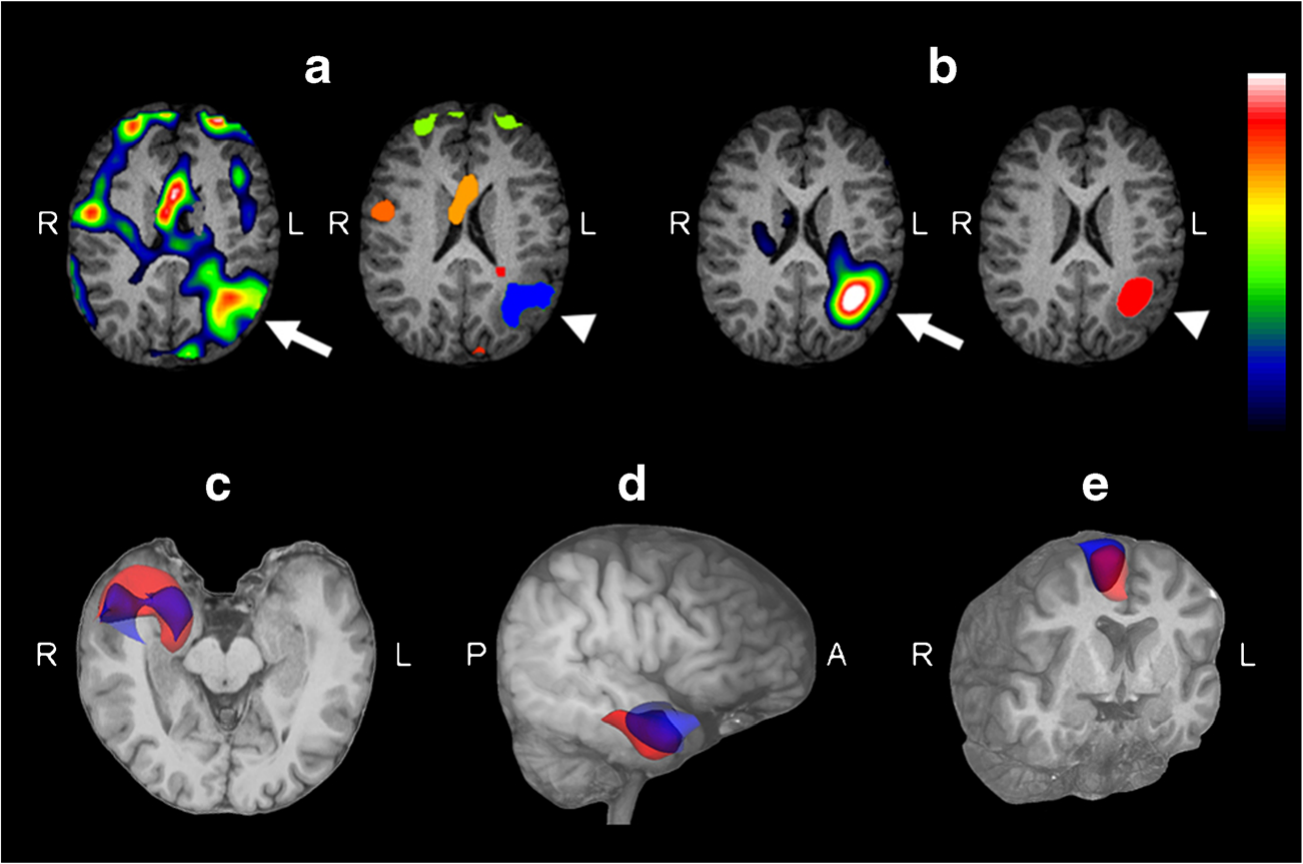
VOI-based analysis. *Top*: Example patient (P23) with a left parietal inferior SOZ shown by SISCOM (**a**) and PISCOM (**b**) (*arrows*) and VOI-based analysis (*arrowheads*). The SISCOM study shows higher indeterminate activity due to propagation, residual count data or artefacts indicated by a greater number of VOIs in the VOI-based analysis. The *colour scale* indicates relative differences between ictal and interictal image values. *Bottom*: Example patients (P01, **c**; P04, **d**; P10, **e**) with a SOZ in the right temporal and right frontal lobes successfully identified concordantly by PISCOM (*red*) and SISCOM (*blue*). Selected SOZ VOIs and MRI images were rendered with 3D Slicer (version 4.6.2) for illustrative purposes. *L* left, *R* right, *A* anterior, *P* posterior

SISCOM showed a higher number of VOIs from indeterminate activity in the 15 concordant successful studies (Table 2) with statistically significant differences compared with PISCOM (mean total numbers of VOIs: 11.3 for SISCOM and 3.5 for PISCOM; *p* = 0.001). Table 2 shows the results of the VOI-based analysis, and the results in an example patient are shown in Fig. 4a.

**Table 2:** Results of VOI-based analysis

Patient no.	Number of VOIs^a^		SOZ volume (cm^3^)		Percent of SOX voxels shared	
	SISCOM	PISCOM	SISCOM	PISCOM	SISCOM shared with PISCOM^b^	PISCOM shared with SISCOM^c^
P01	10	2	8.08	11.82	69.90	47.80
P02	14	5	18.34	16.28	53.38	60.14
P03	6	3	17.90	29.59	84.29	50.97
P04	11	2	8.65	11.10	60.44	47.06
P05	2	4	9.16	6.42	37.34	53.24
P06	23	5	15.34	19.11	64.18	51.53
P07	1	1	6.00	7.13	39.04	32.85
P09	11	8	15.22	9.14	47.27	78.70
P10	13	6	4.92	4.95	66.60	66.29
P11	18	2	8.27	3.98	19.43	40.34
P12	8	3	3.55	2.51	43.88	61.98
P15	5	2	16.21	13.45	67.20	81.02
P18	5	3	2.88	5.75	77.44	38.82
P19	25	6	48.02	50.08	36.38	34.88
P23	18	1	5.28	3.86	43.32	59.28
Mean	11.33	3.53	12.58	13.01	54.01	53.66

^a^Number of SOZ + indeterminate activity VOIs in all 15 patients with concordant successful results

^b^Number of voxels in SISCOM SOZ VOI shared with PISCOM SOZ VOI/total number of voxels in SISCOM SOZ VOI × 100

^c^Number of voxels in PISCOM SOZ VOI shared with SISCOM SOZ VOI/total number of voxels in PISCOM SOZ VOI × 100

SOZ voxel extension delimited by PISCOM and SISCOM was equivalent, with no significant differences between PISCOM and SISCOM SOZ volumes (mean SOZ VOI volume: 12.58 cm^3^ with PISCOM, 13.01 cm^3^ with SISCOM; *p* = 0.67). SISCOM and PISCOM studies in all 15 patients with concordant successful results showed some degree of spatial overlap between the two SOZ VOIs with a mean 54% of shared voxels (range 19–84%, Table 2). The results in example patients are shown in Fig. 4b.

## Discussion

Complex cases of drug-resistant epilepsy can often be studied with MRI, SISCOM and FDG PET, as to date there are no evidence-based guidelines for localization of the EZ. When comparing the two techniques alone, the use of FDG PET in place of interictal SPECT can be justified by the superior sensitivity of interictal FDG PET (85–90% vs. 50%) [9]. Nevertheless, interictal SPECT is required as a baseline study for ictal SPECT subtraction in spite of its low sensitivity. Considering that ictal perfusion SPECT and FDG PET studies provide the most reliable neurofunctional information on the EZ, in the PISCOM technique interictal SPECT is replaced with a reprocessed interictal FDG PET image for subtraction from ictal SPECT. The preliminary PISCOM results in this cohort of patients showed a similar rate of successful SOZ localization to those obtained with SISCOM.

It has been shown that both perfusion SPECT and FDG PET can reveal the regional perfusion or metabolic activity of the brain which are normally closely paired between control subjects and patients with neurodegenerative diseases [28–30]. However, several studies have accurately identified the uncoupling between regional cerebral blood flow and regional cerebral glucose metabolism in epilepsy. This discrepancy may in part be explained by differences in radiotracer behaviour and by the duration of the seizure disorder [13, 15, 18, 20–24].

To evaluate any systematic differences related to the inherent biodistribution of the two tracers, a paired *t* test comparing interictal SPECT and rFDG PET was performed. This analysis showed significant differences exclusively in the cerebellum, midbrain and basal ganglia. Similar to previous reports [23, 31], we interpreted these findings as being related to differences in the biodistribution of the radiotracer within the brain. On the other hand, no significant differences were found at the cortical level, supporting the contention that the two interictal modalities were compatible for comparison and analysis after reprocessing of the FDG PET studies. Hence, we conclude that rFDG PET images are suitable for ictal SPECT subtraction and recommend that cerebellar and subcortical areas normally not involved with the pathogenesis of the SOZ can be excluded with the use of a brain mask.

Even though the concept of registering these two different nuclear medicine techniques is not new [21], to our knowledge there are no studies proposing FDG PET as an effective “baseline study” for ictal SPECT subtraction in SOZ identification. To make ictal SPECT and rFDG PET comparable and suitable for PISCOM subtraction, SPECT images were reconstructed using iterative algorithms including scatter, attenuation and PSF corrections with the aim of obtaining SPECT images with resolution and degradation corrections more similar to those of FDG PET images. In addition, FDG PET images were reprocessed by resampling, filtering and intensity normalization to fit the features of the SPECT images (Supplementary material).

The main result of this study is the good agreement observed between the PISCOM and SISCOM results for SOZ identification in temporal and extratemporal epilepsy, justifying the use of rFDG PET for ictal SPECT subtraction. The SOZ established by PISCOM demonstrated precise spatial coincidence with those the SOZ SISCOM at the sublobar level, with no significant differences in SOZ voxel extension. Furthermore, VOI-based analysis showed important 3D overlap between the SOZ of the PISCOM and SISCOM studies (mean 54% shared voxels), indicating an almost equivalent characterization of the SOZ by the two modalities and supporting the idea that ictal hyperperfusion originates somewhere within the broader dysfunctional hypometabolic areas [10].

**Fig. 5:**
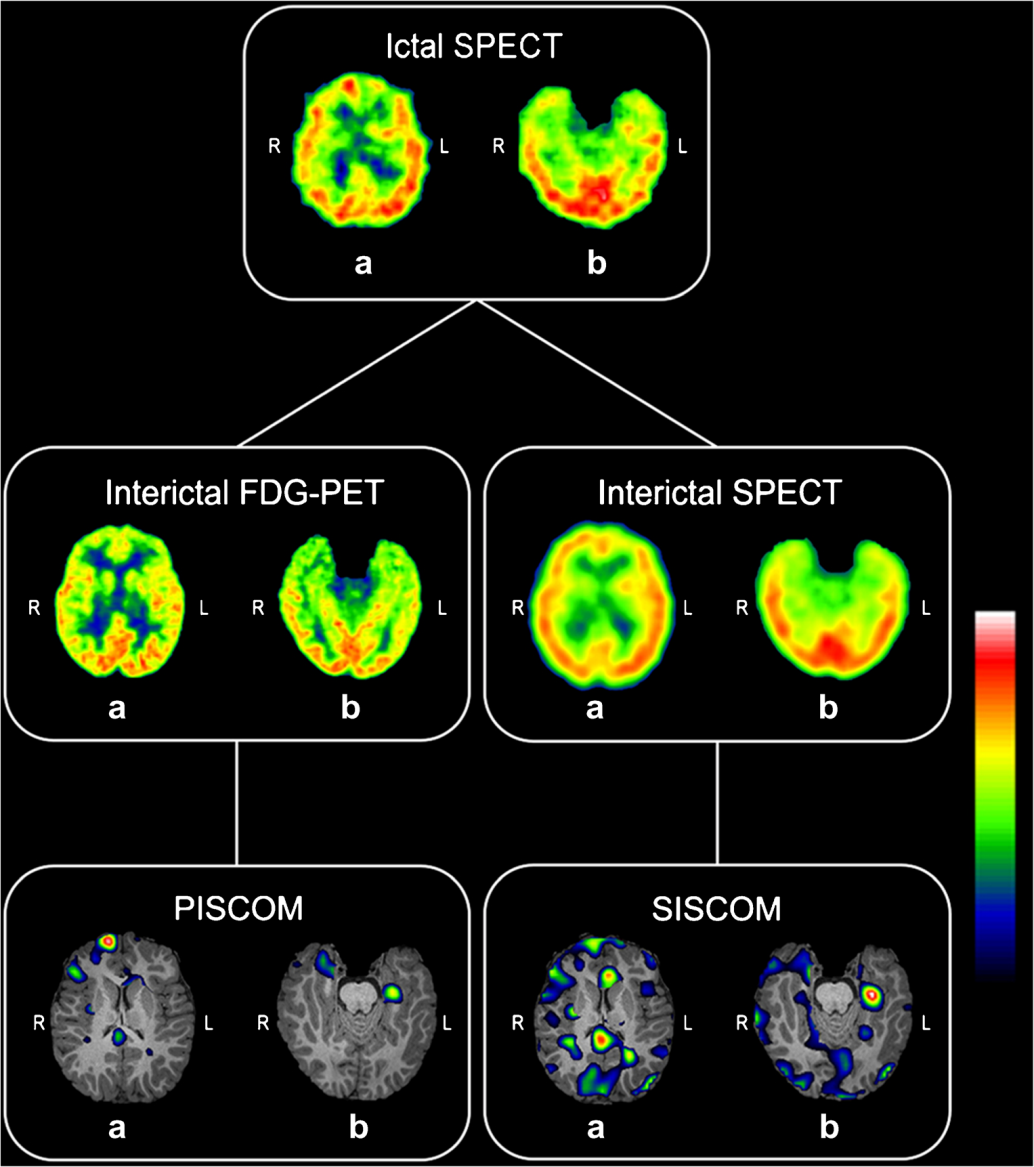
Example patient (P22, a 4-year-old child with a confirmed right frontal epileptogenic zone) showing discordant PISCOM and SISCOM results. Axial ictal and interictal SPECT, original FDG PET, PISCOM and SISCOM images of the brain at the frontal (*a*) and temporal (*b*) lobes. The PISCOM images show successful identification of the SOZ in the right frontal lobule (*a*) while the left medial temporal lobe shows focal uptake with lower activity interpreted as propagation (*b*). The SISCOM images show diffuse uptake in right frontal lobe interpreted as indeterminate activity (*a*), and focal activity in the left medial temporal lobe incorrectly identified as the SOZ (*b*). *Colour scale* intensity values in the SPECT and PET images, relative differences between ictal and interictal image values in the PISCOM and SISCOM images. All images are coregistered with MRI images. *L* left, *R* right

The rate of successful results obtained with PISCOM was slightly higher than that obtained with SISCOM (83% vs. 74%); however, the difference was not significant. In two patients with negative SISCOM studies (P08, P20), PISCOM correctly counterbalanced the intense interictal hypometabolism with normal ictal perfusion, leading to successful identification of the SOZ. A patient showing discordant PISCOM and SISCOM results is shown in Fig. 5.

Subtraction images can contain residual count data in addition to focal areas of increased perfusion [14]. While in the visual analysis propagation activity was observed with both modalities with a similar proportion, in the VOI-based analysis PISCOM showed fewer misleading results due to significantly less indeterminate activity arising from propagation, background or artefacts. Indeed, in two patients (P17 and P22) the high rate of indeterminate activity led to unsuccessful identification of the SOZ by SISCOM. One explanation for these findings is that the contrast between FDG PET hypoactivity and ictal SPECT hyperactivity is higher than that between interictal and ictal SPECT activity. However, it should be noted that PISCOM is not completely free of the problem of indeterminate activity; in one patient (P13) PISCOM misinterpreted the SOZ as propagation activity.

Centres with PET availability and where SISCOM is used as part of routine epilepsy surgery work-up could benefit from the use of PISCOM. Validation of the effectiveness and reproducibility of PISCOM for use in clinical practice in prospective studies would also allow FDG PET to be used as a valid baseline study for subtraction ictal SPECT studies. This would enable the need to perform interictal SPECT studies to be minimized or reserved only for special situations while, at the same time, the FDG PET at its original resolution would provide complementary valuable information for visual and parametric analysis. This scenario could provide important benefits for patients in terms of dosimetry, risk and comfort, especially in those requiring sedation during image acquisition process such as paediatric patients. Furthermore, obviating the need for interictal SPECT may provide significant cost savings in those selected complex cases where ictal SPECT, interictal SPECT and FDG PET are under consideration.

This study had some limitations. It was a retrospective study with a limited number of subjects, especially the subgroup of eight patients with extratemporal epilepsy and the 15 who underwent automatic VOI-based analysis. In addition, the selection criterion of previous resection of the EZ may have introduced bias in overestimating the sensitivity and concordance of all the imaging studies since only those patients with manifest positive and concordant findings would have been considered for surgical treatment. It remains to be determined whether PISCOM is able to detect more subtle changes that could be hidden from the naked eye in a group of patients without surgical intervention.

### Conclusion

This study was an initial proof-of-concept study in the development of a new image processing technique that combines the valuable perfusion and metabolic information provided by ictal SPECT and interictal FDG PET studies. Our results suggest that perfusion and glucose metabolism in the cerebral cortex can be correlated and that PISCOM could be a valid technique for identification of the SOZ, although further studies are needed to validate these results.

## Electronic supplementary material

ESM 1 (DOCX 48 kb)
