# Supplementary material for: PISCOM: a new procedure for epilepsy combining ictal SPECT and interictal PET
Source: Eur J Nucl Med Mol Imaging. 2018 Aug 1;45(13):2358–67. doi: 10.1007/s00259-018-4080-6 (PMC6208811; doi:10.1007/s00259-018-4080-6)
Supplement: Supplementary file 1 — (DOCX 48 kb) [file 259_2018_4080_MOESM1_ESM.docx]

**SUPPLEMENTAL DATA**

**Patients**

We performed a retrospective review of all patients with medically refractory epilepsy undergoing presurgical evaluation and subsequent surgical resection at our centre from 2008 to 2013. The inclusion criteria were: (a) diagnosis of medically refractory epilepsy, (b) the patient had undergone brain MRI, ictal SPECT, interictal SPECT and FDG-PET studies, (c) surgical resection of the suspected EZ and (d) favourable outcome (Engel class I or II) for at least two years after surgery.

The patients were previously evaluated in the clinical work-up to optimally localise the EZ using clinical and neuropsychological examinations, EEG monitoring, and anatomical and functional neuroimaging studies. As described in a previous study ^1^, SISCOM or FDG-PET studies are requested after MRI in patients who are candidates for surgery, especially in cases: 1) with unclear or diffuse video-EEG results; 2) a normal MRI, multiple MRI lesions, or MRI findings discordant to video-EEG; 3) for the evaluation of potential secondary epileptic foci; 4) to better define the limits of the area of the brain to be resected; and 5) for evaluating the functional integrity of the rest of the brain ^2^.

Twenty-three cases with a mean age of 31 years (range: 4-61 years) were retrospectively identified (10 males and 13 females, 18 adults and five children) as fulfilling the inclusion criteria.

Informed written consent was obtained from all patients or their parents or legal guardians, and all the procedures were approved by the hospital Ethics Committee.

**Surgical Treatment and Histological Analysis**

Patient candidacy for surgery was established based on the standard evaluation protocol of the epilepsy unit according to the clinical, electrophysiologic, and neuroimaging data obtained. Lobar or selective cortical resection was performed by neurosurgeons from the epilepsy unit. Histologic analysis of all the brain tissue resected showed 9/23(39%) cases of mesial temporal sclerosis, 9/23(39%) cases of focal cortical dysplasia, two cases of mesial temporal sclerosis associated with focal cortical dysplasia, one case of gliosis, one low grade glioma and one grade I ganglioglioma (Table 1).

**Follow Up**

Postoperative seizure outcome was classified according to the Engel classification: Engel class I (completely seizure-free, auras only, or atypical early postoperative seizures only), Engel class II (≥90% seizure reduction or nocturnal seizures only), Engel class III (>50% seizure reduction), and Engel class IV (<50% seizure reduction). As indicated above, only patients with a favourable outcome (Engel class I or II) for a period of at least two years after surgery were included in the study. All twenty-three patients were classified as Engel I with a median follow up time of 3,41 (ranging from 2 to 5 years) (Table 1).

**Epileptogenic Zone**

The EZ was considered the gold standard, based on surgical resection, histology results and favourable postsurgical outcome, the EZ was located in the temporal lobe in 15/23(65%) patients, with 10/23(43%) presenting lesions in the medial temporal lobe, 3/23(13%) in lateral neocortical temporal lobe and 2/23(9%) in both medial the temporal and the lateral neocortical temporal lobe. Of the remaining 8 patients, the EZ was located in the frontal lobe in 6/23(26%), the insula in 1/23(4%) and the parietal lobe in 1/23(4%) of the patients (Table 1).

**MRI**

All of the 18 adult MRI studies were performed with a 3-Tesla unit (Tim Trio; Siemens, Erlangen, Germany) with a specific epilepsy protocol including the following sequences: coronal T2 FSE (repetition time 5590 ms; echo time 92 ms, 3 mm slice thickness); coronal 3D MPRAGE  (repetition time 2000 ms; echo time 2.98 ms, 0.9 mm slice thickness); coronal  3D  Space Inversion Recovery (repetition time 7500 ms; echo time 324 ms; inversion time 3000 ms, 1.03 mm slice thickness); sagittal 3D FLAIR (repetition time 5000 ms; echo time 396 ms and inversion time 1800 ms, 0.9 mm slice thickness), and axial T2 FSE (repetition time 4640, echo time 99 ms, 3 mm slice thickness). All the coronal sequences were acquired parallel to the long axis of the hippocampus and all the sequences covered the full brain.

The 5 paediatric MRI studies were performed using a 1.5 T unit (Signa Exite; GE Healthcare, Milwaukee, WI, U.S.A.) with a specific epilepsy protocol that included the following sequences: sagittal T1-weighted inversion recovery (repetition time 2,291 ms, echo time 16 ms, inversion time 750 ms, 5-mm slice thickness); coronal proton density (PD)/T2 dual fast spin echo (repetition time 5,240 ms, echo time 29/87 ms, 3-mm slice thickness); coronal 3D magnetization prepared rapid acquisition gradient echo (repetition time 13.3 ms, echo time 5.6 ms, 1.4 mm slice thickness); coronal fluid-attenuated inversion recovery (FLAIR) (repetition time 9,002 ms, echo time 131 ms, and inversion time 2,000 ms, 4 mm slice thickness); and axial PD/T2 dual fast spin echo (repetition time 3,300 ms; echo time 22/88 ms, 5 mm slice thickness). All coronal sequences were acquired parallel to the long axis of the hippocampus and all sequences covered the full brain. Sedation was used in selected cases.

All the MRI studies were interpreted visually by an expert neuroradiologist not blinded to clinical data and other test results.

**SPECT**

Ictal SPECT was performed as part of patient admission for video-EEG monitoring at the epilepsy unit of our centre. Seizure onset was defined as the time of earliest indication of auras or the beginning of rhythmic ictal discharges detected by continuous video-EEG monitoring. The radiotracer was administered intravenously at seizure onset by an experienced nurse trained to manually inject radioactive material while waiting in the EEG technician’s room on the day of the ictal study. Interictal SPECT was performed within the following week. Ictal and interictal SPECT studies were performed with approximately 925 MBq of 99mTc-hexamethylpropyleneamine-oxime (^99m^Tc-HMPAO). The average time from seizure onset to radiotracer injection was 21,52 seconds (ranging from 4 to 46). Sedation was used in selected cases for image acquisition.

Ictal and interictal SPECT images were acquired within two hours after radiotracer injection following the same protocol using a dual-head SPECT imaging system (Infinia^TM^ Hawkeye^TM^ 4; GE Healthcare Milwaukee, WI, USA) with a low-energy high-resolution parallel-hole collimator. The radius of rotation was 14 cm and 60 projections (30 projection per head) were acquired over 360º at 40 s/projection in a 128x128 matrix with a pixel size of 3.32x3.32 mm^2^. In addition to the photopeak imaging window (126keV-154keV) one additional scatter energy window was also acquired (114keV-126keV) for scatter correction.

Reconstruction was performed using the Ordered Subsets Expectation Maximization (OSEM) algorithm ^3^ (10 subsets and six iterations) implemented in STIR library ^4^. The matrix size was of 128x128x54 voxels with a voxel size of 3.32x3.32x3.32mm^3^. Correction for scatter, attenuation and spatially dependent Point Spread Function (PSF) were included in the reconstruction process. Attenuation coefficients were obtained from the CT image obtained from the FDG-PET study, which was then co-registered to both ictal and interictal SPECT images and segmented to separate brain (0.155 cm^−1^) and bone (0.323 cm^−1^) tissues. With the use of the OSEM from STIR rather than the standard software included in the SPECT system, reconstructed SPECT images were obtained with resolution and degradation corrections more similar to those obtained with FDG-PET, thereby facilitating the implementation of the PISCOM methodology.

**PET**

Interictal FDG-PET images were acquired in 3D mode using PET/CT equipment (Biograph; Siemens, Erlangen, Germany). Patients were silent and resting in a dimly lit room during the 40 min following intravenous injection of approximately 5 MBq/kg of ^18^F-Fluorodeoxyglucose, after which images were acquired using a standard 11 min schedule (1 min for CT scan and 10 min for PET). In selected cases, EEG monitoring was performed during FDG-PET image acquisition to confirm interictal state. Reconstruction was performed with the OSEM algorithm (16 subsets and six iterations) using standard software from the gammacamera. All images were reconstructed using a matrix of 128x128x63 with a voxel size of 2.6x2.6x2.4 mm^3^. Sedation was used in selected cases.

SPECT and PET studies were performed within a mean period of 86 days (range 7-201 days). No changes in medication or clinical epilepsy features were documented during this period of time.

**SISCOM**

SISCOM methodology was performed using FocusDET, a previously developed software toolbox for SISCOM analysis ^5^. The main steps to perform SISCOM analysis with FocusDET are: 1) Registration of Ictal and Interictal SPECT studies using rigid body transformation with a local correlation coefficient as a cost function ^6,7^; 2) Intensity normalisation ^6^ and generation of a parametric subtraction image of the relative difference between ictal and interictal image values. The value of voxel i in subtraction image, is calculated as ${SI}_{i}=\left( I_{i}-{II}_{i} \right)\cdot100/{II}_{i}$ where $I_{i}$ and $II_{i}$ are the values in voxel i of the ictal and interictal studies, respectively; 3) Generation of a MRI mask to extract the brain region by performing a semi-automatic segmentation of the MRI ^8^. 4) Co-registration of SPECT and MRI studies with a multi-resolution rigid registration scheme ^9^, and 5) Segmentation of the subtraction image to show only those voxels with values greater than two standard deviations (2SD) that are then fused to the MRI of the patient.

**PISCOM**

With the aim of subtracting interictal PET studies from ictal SPECT, FDG-PET images were reprocessed (rFDG-PET) to assimilate features of the PET and SPECT images as follows: 1) FDG-PET images were resampled to obtain the same matrix and voxel size as those of interictal perfusion SPECT, 2) FDG-PET images were filtered to achieve similar smoothing between SPECT and PET images (the FDG-PET filter was derived from experimental PSF images of the SPECT and the PET acquisition systems), and 3) intensity normalization was applied to FDG-PET images to correct differences in the total number of photons detected. As the loss of resolution that the real image suffers in the acquisition is due to the point spread function (PSF), the FDG-PET filter in step 2 was derived from experimental PSFs obtained from the SPECT and PET systems by using a radioactive point source. Then, Fourier transforms of SPECT and PET PSFs were calculated and Gaussians were fitted to both distributions to avoid the problems derived of high frequency noise. Finally, the filter was obtained as the inverse Fourier transform of the quotient between the Gaussian associated to SPECT and that of PET.

The PISCOM methodology was performed using FocusDET, following the same steps as the SISCOM analysis but replacing interictal SPECT image with rFDG-PET image obtained as explained above. Subtraction image values greater than two standard deviations (2SD) were fused to the MRI of the patient for visual assessment.

**SPM Analysis (rFDG-PET vs interictal SPECT)**

rFDG-PET images were statistically compared with interictal SPECT images using the Statistical Parametric Mapping (SPM8) software (Wellcome Department of Imaging Neuroscience, Institute of Neurology, London) ^10^ to determine any systematic differences related to the inherent biodistribution of the two tracers.

First, interictal SPECT and rFDG-PET images were spatially normalized to a FDG-PET template defined in the Montreal Neurological Institute standard space ^11^. Then, previously normalized interical SPECT and rFDG-PET was smoothed to increase the signal-to-noise ratio. Finally, each rFDG-PET image was compared to its corresponding interictal SPECT image at voxel level using a paired t-test (two sample t-test; group 1 = 23 interictal SPECT images and group -1 = 23 rFDG-PET images). Two contrast levels [1 -1] and [-1 1] were applied to detect any significant regional differences between interictal perfusion and metabolism. For the statistical comparison, an explicit mask based on a brain-level threshold of 0.5 was used to consider only voxels inside the brain (i.e*.* only voxels with an intensity level greater than 0.5 in the brain mask provided by SPM were considered in the statistical comparison). Differences were considered to be significant using the commonly used criteria of p<0.05 with correction for multiple comparisons following the Family Wise Error approach. All significant differences were overlaid on the single subject MRI provided by SPM.

If any significant differences related to the biodistribution of the radiotracer were detected at a subcortical or cerebellar level, a mask based on these findings was created to exclude these areas from the visual and volume-of-interest (VOI)-based analysis of the PISCOM images.

**Visual Analysis**

Each MRI was spatially normalized to the MRI template with a SPM8 normalization tool ^10^. Then, regions from the Automatic Anatomic Labeling (AAL) template ^12^ were placed on each MRI study by applying the inverse transformations of the previously normalized MRI images for better anatomical localisation of the findings.

Interpretation of the PISCOM and SISCOM images was performed separately by 2 experts in nuclear medicine blinded to clinical data and other test results. PISCOM and SISCOM studies were superimposed to basic T1 MRI sequence only to provide anatomical reference. In addition, Automatic Anatomic Labeling (AAL) template was used for better anatomical localisation of the findings. Abnormal findings were defined by visual evaluation of brain areas greater than 2 SDs above the mean activity and SOZ was defined as the cortical area with the greatest subtraction values that could not be explained by propagation, residual count data or artifacts. Presence and localisation of the SOZ and propagation activity in both modalities was reported. Both image modalities of each patient were presented randomly, to ensure that the review of one study would not influence that of the other. In cases of discrepancy between reviewers, consultation and discussion with a third nuclear medicine specialist was done in order achieve consensual agreement.

The PISCOM and SISCOM results were compared with each other and with the EZ localisation. Successful identification of the SOZ must match PISCOM or SISCOM findings with the known EZ location at a sublobar level of accuracy; hence, hemisphere or simply lobar concordance was considered as unsuccessful localisation. Nevertheless, findings located in temporal pole were considered as successful identification of the SOZ regardless of whether the EZ was located in the medial temporal lobe or the lateral neocrotical temporal lobe. When PISCOM or SISCOM failed to identify any focus, the studies were classified as “negative”. In summary, each PISCOM or SISCOM study was classified as “successful”, “unsuccessful” or “negative”.

**VOI-based Analysis**

In order to perform a more objective comparison between PISCOM and SISCOM results, supplementary analysis was carried out in all 15 concordant successful studies. The extension of each SOZ was quantified as well as the amount of indeterminate activity that could hamper the identification of the SOZ in both modalities.

First, the maximum value of the subtraction image in each SOZ identified by PISCOM and SISCOM was determined. Then, VOIs were obtained in each PISCOM and SISCOM study by intensity thresholding (60% of the maximum SOZ value) and clustering (minimum 100 voxels). Thus, each PISCOM and SISCOM study was segmented into several VOIs: one corresponding to the SOZ and the rest of the VOIs that matched thresholds criteria which were considered as indeterminate activity (*i.e*. propagation, background or artifact). Specific algorithms were developed for this task.

The number of VOIs in each PISCOM and SISCOM study was determined to evaluate the amount of indeterminate activity revealed by both modalities. In addition, the number of voxels of each VOI corresponding to the SOZ delimited by PISCOM and SISCOM was quantified to compare the SOZ voxel extension in each patient. Thereafter, the percentage of sheared voxels by the SOZ in both modalities was calculated.

**Statistical analysis**

The proportion of each PISCOM and SISCOM result (“successful”, “unsuccessful”, “negative”) was reported and compared (McNemar test of symmetry). Additionally, global agreement between the results of the two techniques was calculated. The proportion of successful identification of the SOZ by PISCOM and SISCOM was reported with its 95% confidence interval (CI). Variables derived from VOI-based analysis (number of VOIs and SOZ voxel extension) were compared (PISCOM vs. SISCOM) using a paired t-test (a p<0.05 was considered significant). The **s**tatistical analyses were performed using SPSS (version 17.0; SPSS Inc.).

**Supplemental References**

1. Perissinotti A, Setoain X, Aparicio J, et al. Clinical Role of Subtraction Ictal SPECT Coregistered to MR Imaging and 18F-FDG PET in Pediatric Epilepsy. J Nucl Med. 2014;55:1099–1105.

2. Kumar A, Chugani HT. The Role of Radionuclide Imaging in Epilepsy, Part 1: Sporadic Temporal and Extratemporal Lobe Epilepsy. J Nucl Med Technol. 2017;45:14–21.

3. Hudson HM, Larkin RS. Accelerated image reconstruction using ordered subsets of projection data. IEEE Trans Med Imaging. 1994;13:601–609.

4. Fuster BM, Falcon C, Tsoumpas C, et al. Integration of advanced 3D SPECT modeling into the open-source STIR framework. Med Phys. 2013;40:092502.

5. Martí Fuster B, Esteban O, Planes X, et al. FocusDET, a new toolbox for SISCOM analysis. Evaluation of the registration accuracy using Monte Carlo simulation. Neuroinformatics. 2013;11:77–89.

6. Ros D, Espinosa M, Setoain JF, Falcon C, Lomena FJ, Pavia J. Evaluation of algorithms for the registration of 99Tcm-HMPAO brain SPET studies. Nucl Med Commun. 1999;20:227–236.

7. Pavia J, Ros D, Catafau AM, Lomeña FJ, Huguet M, Setoain J. Three-dimensional realignment of activation brain single-photon emission tomographic studies. Eur J Nucl Med. 1994;21:1298–1302.

8. Wollny G, Kellman P, Ledesma-Carbayo M-J, Skinner MM, Hublin J-J, Hierl T. MIA - A free and open source software for gray scale medical image analysis. Source Code Biol Med. 2013;8:20.

9. Studholme C, Hill DL, Hawkes DJ. Automated three-dimensional registration of magnetic resonance and positron emission tomography brain images by multiresolution optimization of voxel similarity measures. Med Phys. 1997;24:25–35.

10. Acton PD, Friston KJ. Statistical parametric mapping in functional neuroimaging: beyond PET and fMRI activation studies. Eur J Nucl Med. 1998;25:663–667.

11. Mayoral M, Marti-Fuster B, Carreño M, et al. Seizure-onset zone localization by statistical parametric mapping in visually normal 18F-FDG PET studies. Epilepsia. 2016;57:1236–1244.

12. Tzourio-Mazoyer N, Landeau B, Papathanassiou D, et al. Automated anatomical labeling of activations in SPM using a macroscopic anatomical parcellation of the MNI MRI single-subject brain. Neuroimage. 2002;15:273–289.
